# Science Possible Selves and the Desire to be a Scientist: Mindsets, Gender Bias, and Confidence during Early Adolescence

**DOI:** 10.3390/socsci6020055

**Published:** 2017-05-31

**Authors:** Patricia Wonch Hill, Julia McQuillan, Eli Talbert, Amy Spiegel, G. Robin Gauthier, Judy Diamond

**Affiliations:** 1 Social and Behavioral Sciences Research Consortium, University of Nebraska-Lincoln, Lincoln, NE 68588-0866, USA; 2 Department of Sociology, University of Nebraska-Lincoln, Lincoln, NE 68588-0324, USA; 3 Department of Psychology in Education, University of Pittsburgh, Pittsburgh, PA 15260, USA; 4 University of Nebraska State Museum, University of Nebraska-Lincoln, Lincoln, NE 68588-0374, USA

**Keywords:** adolescence, bias, gender, identity, mindsets, science, science careers

## Abstract

In the United States, gender gaps in science interest widen during the middle school years. Recent research on adults shows that gender gaps in some academic fields are associated with mindsets about ability and gender-science biases. In a sample of 529 students in a U.S. middle school, we assess how explicit boy-science bias, science confidence, science possible self (belief in being able to become a scientist), and desire to be a scientist vary by gender. Guided by theories and prior research, we use a series of multivariate logistic regression models to examine the relationships between mindsets about ability and these variables. We control for self-reported science grades, social capital, and race/ethnic minority status. Results show that seeing academic ability as innate (“fixed mindsets”) is associated with boy-science bias, and that younger girls have less boy-science bias than older girls. Fixed mindsets and boy-science bias are both negatively associated with a science possible self; science confidence is positively associated with a science possible self. In the final model, high science confident and having a science possible self are positively associated with a desire to be a scientist. Facilitating growth mindsets and countering boy-science bias in middle school may be fruitful interventions for widening participation in science careers.

## 1. Introduction

The gender gap in science persists in many fields despite increases in the participation of women in the paid workforce and 4-year colleges and graduate and professional schools (Ceci et al. 2014). Explanations for this gap include a number of individual, interactional, and institutional mechanisms including gendered socialization, implicit biases, stereotypes, and discrimination (Cheryan et al. 2015; Grunspan et al. 2016; Hill et al. 2010; Moss-Racusin et al. 2012; Xie and Shauman 2003). Evidence suggests that in the United States, in elementary school, both boys and girls have similar levels of interest in science, but by middle school, interest in science among girls has diminished (Andre et al. 1999; Blue and Gann 2008). This disproportionate decline in science interest for adolescent girls compared to boys cannot be due to differences in academic success; on average girls have equivalent or better grades in math and science than boys at every age (Voyer and Voyer 2014).

One compelling explanation for the science interest gap between boys and girls is the complex social, psychological, and developmental processes that happen during adolescence. In particular, there is evidence that gender identity becomes more salient during adolescence (Galambos et al. 1990). For girls compared to boys, greater gender identity salience can result in lower self-esteem, and reduced confidence across many social and psychological domains (Orenstein 2013), including confidence in science and math (Heaverlo et al. 2013). Biased self-assessments may also emerge from implicit and explicit biases in the U.S. and other western European countries where there is a widely held cultural belief that boys are better at science (and math) than girls (Nosek et al. 2009; Cai et al. 2016). Because of the bias towards boys as science kinds of people, girls may not perceive their gender identity as compatible with a science identity (Nosek et al. 2002; Nosek and Smyth 2011). Considerable research has been focused on how to close the “science identity gap” for girls, as well as other underrepresented minorities in science (Archer et al. 2010, 2012, 2013; Barton et al. 2013; Kozoll and Osborne 2004).

The challenges that some girls experience combining “girl” and “science” identitiesmay be influenced by whether or not valued abilities are viewed as innate or attained through effort (Blackwell et al. 2007; Levy et al. 1998). Indeed, research in adult populations show that at least some of the science gender gap can be explained by mindsets about intelligence (whether it is fixed or whether it can be developed through effort), and gendered assumptions about boys’ presumed “innate brilliance” compared to girls’ presumed “hard work” (Leslie et al. 2015).

The extent to which stereotypes about girls and science, and mindsets about intelligence influencing science identity, warrants further investigation. Because science career aspirations begin forming in early adolescence (Tai et al. 2006), one way that researchers have explored the relationship between science identity and science career aspirations is by assessing youth science possible selves (Packard and Nguyen 2003), or the perception that they may someday become a scientist (Oyserman et al. 2006). There are no studies of middle school aged youth that have simultaneously included measures of mindsets, boy-science bias, science confidence, and science possible selves, yet there are reasons to expect that each of these concepts contributes to the desire to be a scientist.

In this study, we assess science possible selves, and the desire to be a scientist in a sample of 529 adolescents in a U.S. Title I (high poverty) middle school. Using developmental theories about gender identity, mindsets, and science possible selves, we assess multiple hypotheses about how gender, grade level, and mindsets are associated with boy-science bias, science confidence, science possible selves, and the desire for a science career.

### 1.1. Gender Identity in Early Adolescence

Adolescence is an important time in the life course. Children begin the social and physical transition to adulthood, and in so doing explore, affirm, or cast aside identities (Eccles et al. 1997; Eckert 1989; Eder 1995). Gendered identities are also “under construction” in early adolescence, when youth are “trying on gender” and other identities as they imagine futures compatible with salient identities, particularly related to gender (Williams 2002).

According to the gender intensification hypothesis, gender identities become more relevant in adolescence, and the intensification contributes to lower self-esteem and reduced mental health for girls (Galambos 2004; Pettitt 2004). Similarly, confidence drops more for girls than boys during adolescence in many areas of life, including in science and math (Orenstein 2013). In school contexts, gender intensification may be explicitly or implicitly endorsed by peers and significant adults (Eder 1995; Adler et al. 1992; Thorne 1993). A classic ethnographic study by Eder (1995) showed that middle school is a time when girls more often become objectified and sexualized, and where social status for girls is often based on physical appearance, relationships with boys, and friendships with girls, compared to an emphasis on achievement for boys (Eder 1995). Bullying is more common during middle school than elementary and high school (Olweus 2013), and sexual harassment of adolescent girls is widespread (Leaper and Brown 2008).

In addition to the overt sexualization and objectification of many girls during puberty, many girls also face academic sexism (Archer et al. 2013; Banchefsky et al. 2016). Academic sexism involves actions that discourage girls from participating in areas deemed as “male”, such as science, math, and computers (Leaper and Brown 2014). In a study of 600 girls, Leaper and Brown (2008) found that 52% of the sample reported some form of academic sexism related to math and science, with the majority perpetrated by peers, but also reportedly from parents. If being desirable, feminine, and sexy is perceived as incompatible with interest and achievement in science, girls may distance themselves from science and also fail to form friendships around science (Archer et al. 2013; Banchefsky et al. 2016). Peer influence can increase or decrease academic achievement, positive identities, and overall well-being (Crosnoe and McNeely 2008; Crosnoe et al. 2008; Leaper et al. 2012). Friendship groups are highly segregated by gender (Shrum et al. 1988). Altogether, these social and cognitive processes and biases may influence science aspirations differently for boys and girls (Gauthier et al. 2017).

### 1.2. Mindsets and Gender Stereotypes

There is compelling evidence that implicit theories about the malleability of traits (i.e., mindsets) can foster or inhibit the development of possible future selves (Levy et al. 1998; Levy and Dweck 1999; Stroessner and Dweck 2015). According to Dweck (2006), people with a growth mindset believe that abilities can be developed. With a fixed mindset, people believe that intelligence or talent are simply fixed traits that they were either born with, or not. People with fixed mindsets focus on documenting intelligence or talent instead of developing intelligence and talents (Dweck 2006). There is evidence that a fixed mindset might emerge from fundamental cognitive processes that help people make sense about the world, but can also lead to errors about the world (Bigler and Liben 1993).

The process of overly simplistic categorizing can lead to inflating differences between groups and ignoring variation within groups, resulting in stereotyping and biases (Master et al. 2012). One common type of error in reasoning that leads to gender stereotypes is called psychological essentialism, or the belief that people naturally possess certain traits based on group characteristics (Stroessner and Dweck 2015; Cimpian and Salomon 2014). Gender essentialism is the belief that differences between boys and girls are natural or innate (based in biology) and that they cannot be changed (Eidson and Coley 2014). This is because if boys are seen as naturally or effortlessly brilliant, and science requires brilliance, then fixed mindsets about intelligence and essentialist mindsets about gender may lead to a science-gender bias favoring boys, and disfavoring girls.

For girls, a boy-science bias might contribute to the pattern of more girls than boys becoming disinterested in science, and may result in a lower likelihood of having a science possible self and/or a desire to be a scientist in middle school for girls more than boys. Conversely, for boys, in group-favoritism (favoring those who belong to your social group) and intergroup biases (disfavoring those not in your social group), may translate into a boost from boy-science bias, resulting in a stereotype lift effect for science possible selves and a desire to be a scientist (Walton and Cohen 2003; Tajfel and Turner 2004).

Although many people perceive that gender stereotypes and biases have disappeared, recent research (2014) shows that there are similar levels of gender stereotypes among contemporary college age youth as in 1980 (Haines et al. 2016). Even though there is evidence that actual explicit gender stereotypes persist, they are likely underreported in surveys due to social desirability.

### 1.3. Science Confidence and Science Possible Selves

In the United States there is a strong belief that youth can choose any career; therefore it can be popular to blame under-representation of women in science and engineering professions on personal preference rather than social structural inequality (Rosenbloom et al. 2008). Charles and Bradley (2009) argue that the higher standard of living in the United States, combined with implicit gender biases about science, contribute to many youth “indulging our gendered selves” when ‘choosing’ career paths. Research suggests, however, that more than simply reflecting an individual’s abilities, career paths are also shaped by social identities and cultural beliefs about who we are, and where we fit in, perhaps even more than what we are good at (Correll 2001, 2004; Cech 2013).

Our identity, or how we see our self, is a social construction; it is a product of shared social interactions and cognitive processes related to social and self-categorizations (Tajfel and Turner 2004; Burke and Stets 2009; Turner et al. 1987). These conceptions of the self are dynamic and are based on our experiences of the social world, including our self-appraisals and reflected appraisal from significant others (e.g., parents, teachers, and peers) (Bouchey and Harter 2005; Gunderson et al. 2012). Our identities are not socially constructed in a vacuum, but are formed within larger social structures and within social institutions (like schools) that are also gendered (Charles and Bradley 2009; Acker and Oatley 1993; Connell 2014; Ridgeway 2009; Risman 2004). Therefore, given these social and institutional contexts, these self-appraisals may be biased or inaccurate and may vary by gender (Correll 2001). At the college level, women’s biased self-assessments and perceptions of a lack of “fit” can impact women’s persistence in some Science, Technology, Engineering, and Math (STEM) fields, (e.g., engineering and computer science) (Cech 2013; Cech et al. 2011; Master et al. 2015). In international studies on adolescent education and achievement, for youth in some high achieving countries, researchers find a negative relationship between student achievement and self-concept; the better students do, the lower they rate their own abilities (Wilkins 2004). In a national study of eighth grade girls in the U.S., researchers found that these biased self-assessments in science are more likely for girls than for boys (Riegle-Crumb et al. 2011); this phenomenon is sometimes referred to as the “confidence gap” (Orenstein 2013).

Identities shape our actions and choices, plus they influence our commitment to pursuing future goals. Therefore, these emerging identities in adolescence are important for many long-term social, emotional, and career outcomes (Eccles et al. 1997; Schwartz et al. 2015). Adolescents make choices about who they are friends with, what activities they pursue, and in high school, what classes to take, in order to validate their identities and to maintain their self-esteem (Barton et al. 2013; Barber et al. 2005; Cast and Burke 2002). Adolescence is also a time when many youth are asked what they want to be when they grow up. Images of who youth might be in the future are referred to as possible selves (Markus and Nurius 1986). Possible selves can be either negative or positive and a possible self that a person finds plausible will affect their current behavior and choices (Oyserman et al. 2006).

A science possible self, or the belief that you might be able to become a scientist someday, is one outcome of emergent science identities during adolescence (Buday et al. 2012). A student who believes that they might be a successful scientist in the future is more likely to express interest in scientific endeavors, excel in science classes, and to form friendships around science activities (Robnett and Leaper 2013). Indeed, the social aspect of science is often overlooked, even though we know that social interactions, validation, and recognition are important for identity (Carlone and Johnson 2007). In a longitudinal study of 41 high school girls who transitioned into college, researchers found social support and mentoring to be important predictors of science career-related possible selves (Packard and Nguyen 2003). Lips (2004) found that college and high school age women were much less likely to have science possible selves compared to men, and that college-aged women saw even less science possibility than high school women, indicating that science pathways constrict more for women than men over time (Lips 2004). In a more recent study, Buday, Stake, and Peterson (2012) found that for both boys and girls, social support was crucial to having a high science possible self, but did not find a gender differences in science possible selves (Buday et al. 2012).

The aforementioned studies all explore science possible selves, but had small sample sizes and were not representative of a general population of students. In addition, these studies have consisted of youth who had been identified as having science and math aptitude and been enrolled in specific science focused programs based on that aptitude and interest. In addition, no studies simultaneously examine mindsets, boy-science bias, science confidence, science possible selves, and the desire to be a scientist. Middle school is a time for early career exploration when science career preferences may emerge, strengthen, or for some, diminish (Tai et al. 2006; Dabney et al. 2015, 2012). Clearly, we need more investigation of identity formation, science possible selves, and youth trajectories in science among boys and girls to understand how science possible selves may be associated with science career aspirations more broadly (Buday et al. 2012).

### 1.4. Current Study

Our goal is to add to the emerging understanding of the origins of gender gaps in science interest by modeling the sources of the gap using a series of multiple logistic regression models. We use a sample of 529 middle school youth in a midsized Midwestern middle school to first assess how middle school youth differ on key focal science attitudes and beliefs by gender. We then assess whether gender, grade level, or mindsets are associated with having a boy-science bias after adjusting for social capital and racial/ethnic minority status. Next, we assess the extent to which gender, grade level, mindsets, and boy-science bias are associated with science confidence after controlling for self-reported grades, social capital, and racial/ethnic minority status. Theories of stereotype formation indicate that biases among boys and girls may be associated with boy-science bias and science confidence differently by age, therefore we estimate interaction by gender and grade level. In addition, theories about in-group bias and stereotype lift suggest that the association between boy-science bias and science possible selves should be gender specific, therefore we estimate an interaction by gender.

### 1.5. Hypotheses

H1: Boys will have higher boy-science bias, science confidence, science possible self, and a desire to be a scientist than girls. Boys and girls will not differ on science grades, fixed mindsets, or essentialist mindsets.

H2: For all youth, including both boys and girls, fixed or essentialist mindsets will be associated with having a boy-science bias, after controlling for minority status and social capital variables.

H3a: For girls, but not boys, boy-science bias will vary by grade level; girls in lower grade levels will have less boy-science bias than girls in higher grade levels, after adjusting for mindsets, and controlling for self-reported grades, minority status, and social capital variables.

H3b: For girls, but not boys, we expect that science confidence will vary by grade level; girls in lower grade levels will have higher science confidence than girls in higher grade levels, after adjusting for mindsets, boy-science bias, and controlling for self-reported grades, minority status, and social capital variables.

H4a: For girls, but not boys, boy-science bias will be associated with a lower likelihood of a science possible self, after adjusting for mindsets, science confidence, and controlling for self-reported grades, minority status, and social capital variables.

H4b: For boys, but not girls, boy-science bias will be associated with a higher likelihood of a science possible self, after adjusting for mindsets, science confidence, and controlling for self-reported grades, minority status, and social capital variables.

H5: For all youth, higher science confidence and higher science possible self will be associated with a desire to be a scientist, after adjusting for mindsets, boy-science bias, and controlling for self-reported grades, minority status, and social capital variables.

## 2. Materials and Methods

We used SPSS version 22, and t-tests and chi-square tests to compare means and proportions for all theoretical variables by gender. Next, we show correlations between variables using a Pearson’s r correlation to assess for multicollinearity and to assess bivariate relationships between key theoretical variables. Finally, we use multivariate logistic regression to estimate associations with boy-science bias, science confidence, science possible selves, and the desire to be a scientist. Because of important prior work on the underrepresentation of some race/ethnic minority groups and elitism in science, we control for race/ethnic minority status and social capital in all models (Catsambis 1995; Hazari et al. 2013).

### 2.1. Participants

The data collected for this study are from Wave III of the *Study of Science Identity in Middle School*, (collected in January 2015). All sixth, seventh, and eighth grade students enrolled in science classes at a Title I (high poverty) Middle School in a mid-sized Midwestern city were asked to participate in the survey. All parents or guardians of potential participants were notified of the opportunity to participate in the survey with an automated phone call and email, and were provided a form to opt their child out of the study if desired. These forms were available in English, Spanish, Vietnamese, and Arabic. Of the 645 students at the school, 95% (610) were enrolled in a science class. Those who were not were either suspended or were placed in a low proficiency English Language Learner (ELL) classroom instead of a science classroom. Of those eligible to participate, 87% (533) chose to participate in the survey, 529 of which we have complete data for all analytic variables. Institutional Review Board (IRB) approval was obtained for this study prior to participation.

Because this is a study of a single school, we use caution in generalizing the findings. This school is demographically diverse. A high proportion of youth come from racial/ethnic minority groups (69.9%), and a large proportion of youth receive free and reduced lunch (78%). Not only can we not generalize, the gender dynamics in this school may be different than in schools with higher socio-economic status (SES) and that are less diverse (Armstrong et al. 2014; Hamilton and Armstrong 2009). Even with this limitation, this research can provide insights into gender, identity development, and science career aspirations during middle school years, and suggestions for valuable further exploration of this critical developmental time.

### 2.2. Measures

To assess the extent to which youth have a desire to be a scientist when they grew up, we asked them, “How much, if at all, do you want to be a scientist?” (1 = A lot, 2 = Some, 3 = A little, 4 = Not at all). We dichotomized this variable so that wanting to be a scientist “A lot” = 1 (7.3%) and all other categories have a value of 0.

We operationalized science possible selves in order to take into account that many youth in early adolescence might see a science career path as a possibility, but might favor another career path more (Archer et al. 2014). For middle school students, asking how much they want to be a scientist might not capture their perception of how open a science path is to them. For example, even students who want to be a famous musician, actor, or athlete might still see science as a possible path. Therefore, we measure a science possible self with the following item: “For this question, let’s pretend you want to be a scientist when you grow up. Which of the following best describes you?” (1 = I could become a scientist, 2 = I might be able to become a scientist, 3 = I probably could not become a scientist, 4 = I could not become a scientist, and 5 = I don’t know). We dichotomized this variable so that those that reported “I could become a scientist” have a value of 1 (23.1%), and all other categories are a zero.

To measure science confidence, students were asked, ‘How good are you at science?’ (1 = Poor, 2 = Fair, 3 = Good, 4 = Excellent). We dichotomized this variable so that those who report they are “Excellent” at science = 1 (20.2%).

To measure explicit boy-science bias we asked the question “Do you think boys or girls are better at science?” The response categories are similar to a measure of explicit science and math gender stereotypes used in other studies that provide a category in which boys and girls are the same, indicating no stereotype (Nosek et al. 2009, 2002; Cai et al. 2016). We dichotomized the responses into those who think girls are better at science, and those think boys and girls are the same at science (boy-science bias = 0), compared to those who think that boys are better at science (more boy-science bias = 1). Our coding reflects the dominant cultural stereotype in the U.S.; that boys are better at science than girls. Approximately 16% of all students report that boys are either a little or a lot better at science than girls.

We assess the extent to which youth have fixed mindsets based on an item from Dweck (Blackwell et al. 2007) that we modified for readability based on the young age of our sample. Students were asked how much they agree with the following statement, “You can learn new things, but you can’t really change how smart you are.” This variable had a range from 1–5 where 1 = Strongly Disagree, 5 = Strongly Agree. The mean is 2.5 (S.D. = 0.05). We also developed a measure guided by the theory of mindsets to assess essentialist mindsets, “Some people are just naturally good at things (like sports, science or music) and will never have to work hard at them.” This variable had a range from 1–5 where 1 = Strongly Disagree, 5 = Strongly Agree. The mean is 2.7 (S.D. = 0.06)

Science grades were self-reported; we asked students “What grades do you usually get in science classes?” (1 = Mostly below C’s, 2 = Mostly C’s, 3 = Mostly B’s and C’s, 4 = A mix of A’s, B’s, and C’s, 5 = Mostly B’s, 6 = Mostly A’s and B’s, 7 = Mostly A’s). The mean is 5 (S.D. = 0.07).

We measure social capital using two variables, the number of books in the home and college expectations. We asked students, “About how many books do you have in your home?” (1 = 0–10 books, 2 = 10–100 books, 3 = Over 100 books). Approximately 22.7% of students reported 0–10 books, 53.1% reported 10–100 books, and 24.2% percent reported more than 100 books at home. Students were also asked, “How likely is it that you will be able to go to college?” (1 = Not at all likely, 1.5 = I don’t know, 2 = A little Likely, 3 = Somewhat Likely, 4 = Very likely). The mean is 3.4 (S.D. = 0.83). We chose to use books in the home because youth are often unable to report accurately on parental income, and this is a widely used measure for youth assessing academic outcomes and achievement internationally (Provasnik et al. 2012). In addition, differences in career aspirations by social class have also been associated with different college expectations dependent upon social class (Grodsky and Riegle-Crumb 2012; Buchmann and DiPrete 2006; Legewie and DiPrete 2012).

We include race/ethnic minority status as a control variable. Students were asked, “What is your race/ethnicity? You can mark more than one answer.” Response categories were; “Black/African American,” “Latino/Hispanic,” “Middle Eastern/Arabic” “White,” “Asian,” “Native American,” “Pacific Islander,” “Mixed,” and “Other,” with space to write in any other race/ethnic group. Approximately 30% of the respondents were white only. Latino (23.5%) and middle eastern (7%) are ethnic categories, so any student who marked these, no matter what other race category they marked, were included in the under-represented race/ethnicity minority category. About a fifth of the sample self-identified as black, 6% Asian, 6.2% Native American, and 3% other. We dichotomized the responses into minority = 1 (69.9%) or not minority = 0.

## 3. Results

### 3.1. Bivariate Results

We provide bivariate results by gender (shown in Table 1) to assess hypothesis 1, whether boys and girls differ on the desire to be a scientist, science possible selves, science confidence, and boy-science bias. For continuous variables, we used t-tests and for categorical variables we used chi-square tests. We find evidence to support hypothesis 1. Compared to girls, a higher proportion of boys want to be a scientist (10% vs. 5%, *p* = 0.048), and believed that they could become a scientist if they wanted to (26% vs. 19%, *p* = 0.031), and reported that they were ‘Excellent’ at science (24% vs. 16%, *p* = 0.014). More boys than girls believed that boys are better at science (had boy-science bias) (22% vs. 11%, *p* = 0.003). There were no significant differences between boys and girls on reported science grades, fixed mindsets, essentialist mindsets, or college expectations. There are no differences by gender for the control variables, minority status, and books in the home.

Table 2 shows the bivariate Pearson’s *r* correlation matrix for the theoretical and control variables. The strongest associations in the matrix are between science grades and science confidence (*r* = 0.40, *p* < 0.001), science possible selves and the desire to be a scientist (*r* = 0.37, *p* < 0.001), and science confidence and science possible selves (*r* = 0.35, *p* < 0.001). Girls, minorities, and youth with less social capital have lower science possible selves and lower science confidence than boys, non-minorities, and those with higher social capital. Youth with lower grades and less social capital are also more likely to hold a boy-science bias. Grade level has a negative association with science grades, indicating that science grades are lower for youth in 8th compared to 6th grade.

Youth with fixed mindsets are less likely to have a science possible self (*r* = −0.19, *p* = 0.001), more likely to have boy-science bias (*r* = 0.14, *p* < 0.021), lower science grades (*r* = −0.28, *p* = 0.001), and lower college expectations (*r* = −0.10, *p* = 0.018). Also, youth with essentialist mindsets were more likely to have fixed mindsets (*r* = 0.26, *p* < 0.001), a higher likelihood of having a boy-science bias (*r* = 0.10, *p* < 0.05), had lower science grades (*r* = −0.09, *p* = 0.041), and lower college expectations (*r* = −0.10, *p* = 0.18) than youth without essentialist mindsets.

### 3.2. Multivariate Results

Table 3 shows the results of a series of logistic regressions with the likelihood of boy-science bias (Model 1 and 2) and science confidence (Model 3 and 4) as outcomes to test hypothesis 2, hypothesis 3a, and hypothesis 3b. Table 4 shows the results of a series of logistic regressions with the likelihood of a science possible self (Model 1 and 2), and the desire to be a scientist (Model 3) as outcomes to test hypothesis 4a, hypothesis 4b, and hypothesis 5.^1^ All ordinal variables are mean centered to adjust for multi-collinearity, to more easily interpret the constant/intercept, and to solve for and plot significant interactions.

### 3.3. Boy-Science Bias and Science Confidence

Table 3, Model 1 shows the associations of grade level, gender, and fixed and essentialist mindsets with the likelihood of having a boy-science bias. The results show that girls are less likely to have a boy-science bias (β = −0.75, *p* = 0.004) after adjusting for other variables in the model. Additionally, we find evidence that partially supports hypothesis 2; fixed mindsets, but not essentialist mindsets, are associated with boy-science bias (β = 0.24, *p* = 0.021). Youth with fixed mindsets are more likely to have boy-science bias than youth without fixed mindsets.

**Figure 1:**
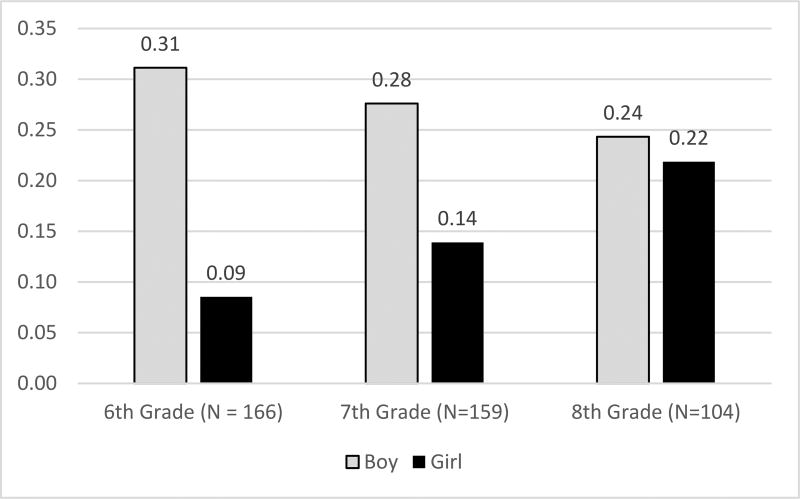
Predicted Proportion with a Boy-Science-Bias by Grade and Gender, adjusted for control variables.

In Model 2, to test for hypothesis 3a, we include a measure of the interaction of gender by grade level. There is support for hypothesis 3a because the interaction is significant on boy-science bias (β = 0.72, *p* = 0.027). The main effect for grade level is not significant, indicating that in the adjusted model, for boys, boy-science bias does not differ by grade level. Figure 1 shows the predicted probabilities for boy-science bias by gender and grade-level.

Figure 1 shows the predicted proportion with a boy-science bias for 6th, 7th, and 8th grade boys and girls. Among boys, there is a slight, non-significant decline in the proportion with a boy-science bias from 6th (31%) to 8th (24%) grade. The proportion of girls with a boy-science bias is largest for 8th grade girls (22%), smaller for 7th grade girls (14%), and smallest for 6th grade girls (9%). The difference between boys and girls is largest in 6th grade (22%).

Table 3, Model 3 shows the multivariate logistic regression model for science confidence. After adjusting for control variables, effects of gender and grade level are only marginally significant. There were trends that were marginal on science confidence for girls and by grade level. Girls have lower science confidence than boys (β = −0.47, *p* = 0.067). Higher grade level is associated with higher science confidence (β = 0.26, *p* = 0.095). Similar to the bivariate model, there is no significant relationship between boy-science bias, fixed mindsets, essentialist mindsets, and science confidence. The only significant association is between self-reported grades and science confidence (β = 1.07, *p* < 0.001). Although minority status and social capital had significant associations with science confidence in the bivariate model, they are no longer significant in the full multivariate model. In Model 4, we tested an interaction between gender and grade to assess hypothesis 3b; that boys’ confidence would not vary by grade level, while girls’ science confidence would be lower as grade level increase. We failed to find support for this hypothesis.

### 3.4. Science Possible Self and the Desire to Be a Scientist

Table 4, Model 1 shows the multivariate logistic regression results for science possible selves. Results show that grade level has a positive association with science possible selves (β = 0.33, *p* = 0.025); therefore being in a higher grade is associated with higher science possible selves compared to being in a lower grade. Similar to the bivariate level, girls have lower science possible selves than boys (β = −0.40, *p* = 0.096), although the effect is only marginal after adjusting for controls. Science confidence has a significant association with science possible selves (β = 1.49, *p* < 0.001), followed by boy-science bias (β = −1.00, *p* < 0.018). Youth with fixed mindsets have lower science possible selves (β = −0.28, *p* = 0.013) than youth without fixed mindsets. In contrast, youth with college expectations have higher science possible selves (β = 0.57, *p* = 0.002) than youth without college expectations. In Model 2, we test an interaction of gender and boy-science bias to assess hypothesis 4a and hypothesis 4b. The results do not support hypotheses 4a and 4b; the association of boy-science bias with science possible selves does not differ for boys and girls.

Finally, we assess the relationship between all previous theoretical variables and the desire to be a scientist (Model 3). We find support for hypothesis 5; high science confidence is associated with higher odds of having a desire to be a scientist (β = 1.48, *p* = 0.001). Having a science possible self was also associated with higher odds of having a desire to be a scientist (β = 2.42, *p* < 0.001).

## 4. Discussion

This study provides a comprehensive analysis of how a fixed mindset and essentialist mindsets are associated with boy-science bias, science confidence, science possible selves, and the desire to be a scientist in a large sample of early adolescents in a U.S. middle school. Several findings are noteworthy. First, despite relatively high proportions of youth with high science confidence and high science possible selves (about 25%), very few say they want to be a scientist “A lot“; only approximately 7% in the whole sample. Although almost twice as many boys desire to be a scientist in the bivariate model (10% compared to 5%), gender differences are not significant in the adjusted model, indicating that gender gaps in science are related to differences in science possible selves and science confidence among boys and girls. Indeed, science confidence and a science possible self were both independent predictors of a strong desire to be a scientist.

While the only significant association with science confidence was self-reported grades in science in the multivariate models, science possible self was associated with many more variables. Girls, youth with a boy-science bias, and with a more fixed mindset were less likely to have a science possible self, while grade level, science confidence, and college expectations were associated with a higher likelihood of a science possible self. Although we do not find that a boy-science bias has a direct association with youth desire to be a scientist, it has a negative association with a science possible self for boys and girls. Therefore, higher levels of boy-science bias in 8th grade girls compared to 6th grade girls may explain at least some of the emergent gender gap in science during early adolescence.

Although we hypothesized that a boy-science bias would be associated with lower odds of having a science possible self for girls, and possibly higher for boys, we found it was associated with having a lower likelihood of a science possible self for boys and girls. Theories about in-group biases led us to hypothesize that a boy-science bias might give boys’ science possible self a boost or lift (Walton and Cohen 2003). We therefore plan to conduct more research to understand how boy-science bias could operate in the same way for boys and girls. It may be that some boys have unrealistically high expectations of themselves, which do not match their perceived ability. It might also be the case that strong in-group favoritism may be protective against low self-appraisal/self-esteem related to underachievement or disinterest in science.

Science confidence had robust associations with having a science possible self and desire to be a scientist, and the trend was that girls, on average, had lower confidence. The associations, however, were only marginal in the multivariate model. We also did not find evidence that confidence differs by grade between boys and girls in adolescence, although youth in higher grade levels had more confidence than in lower grades, exploratory analyses suggest this is driven by boys’ confidence increasing relative to girls, and not girls’ confidence decreasing. Science confidence has significant associations with having a science possible self and the desire to be a scientist. Efforts to increase science confidence among youth and longitudinal follow-up could better identify if such efforts could help boys and girls maintain science interest and career aspirations.

Fixed mindsets about intelligence were associated both with a boy-science bias, and with science possible selves in multivariate models. Essentialist mindsets were only associated with boy-science bias and fixed mindsets in the bivariate models, and were not associated in the multivariate models. These intriguing findings indicate that youths’ beliefs about intelligence, whether it is fixed or malleable, are associated with boy-science bias and science possible selves. Therefore interventions to foster growth mindsets and science possible selves could maintain, widen, and broaden interest and persistence in STEM (Leslie et al. 2015; Meyer et al. 2015).

As with all research, there are important limitations to the generalizability of these results. First, this is a study of a single school; schools can vary considerably, and variables associated with adolescent culture might influence gender identity and gender stereotypes (Legewie and DiPrete 2014). Second, this study is cross sectional. Although we interpret the differences between sixth, seventh, and eighth graders, it is possible that the differences we see are cohort effects and not developmental effects. Although theory and empirical research supports that there is likely a developmental change in boy-science bias views, we cannot conclude from these findings that the differences we see by grade level are necessarily developmental. We would need a longitudinal study to assess how these attitudes change over time.

Our measures of social capital are limited, and recent research linking social capital with “science capital” and science career aspirations indicates that this is likely an important aspect of science career aspirations and science identity for adolescence (Archer et al. 2015). Future studies should consider including measures of socioeconomic status, and in particular, “science capital” including exposure to scientists, and exposure to science media, and informal science outside of schools (Archer and DeWitt 2015). The association between mindset, stereotyping, and science possible self is another possible rich avenue for exploration. Although much of our emphasis is on reducing the relevance of gender for STEM engagement, it might be worth exploring if in-group bias favoring science is protective against gender stereotypes. A longitudinal study of youth from 4th through 8th grade to assess change in mindsets, boy-science bias views, and science possible selves might unpack how these constructs change over time for girls, and lead to possible promising areas for interventions.

Overall there is evidence that decreasing fixed and increasing flexible mindsets has the potential to increase science possible selves and the desire for a job in science. Efforts to help youth learn about how they learn and the possibilities for learning (i.e., that they do not have to be born a scientist), seem promising for increasing interest in science careers. Attempts to de-gender science or to make science gender neutral may also be worthwhile because boy-science bias was associated with lower science possible selves for boys and girls. Finally, providing more concrete information about possible science careers could help youth to imagine a possible self with work involving science.

## Figures and Tables

**Table 1:** Bivariate Descriptive Statistics by Gender

	Boys (*N* = 284)		Girls (*N* = 245)		*sig.*

	Mean/Proportion	*S.D.*	Mean/Proportion	*S.D.*	
Desire to Be a Scientist	0.10		0.05		*
Science Possible Self	0.26		0.19		*
Science Confidence	0.24		0.16		*
Boy-Science Bias	0.22		0.11		**
Fixed Mindset	2.52	1.23	2.54	1.21	n.s.
Essentialist Mindset	2.71	1.31	2.61	1.27	n.s.
Science Grades	5.09	1.59	4.06	1.65	n.s.
Minority	0.69		0.70		n.s.
College Expectations	3.39	0.84	3.47	0.82	n.s.
Books in the home (0–10 reference)	0.24		0.25		n.s.
10–99 books	0.52		0.54		n.s.
100+ books	0.24		0.21		n.s.

Note:

*** p < 0.001,

** p < 0.01,

* p < 0.05,

n.s. = not significant.

**Table 2:** Bivariate Correlation Matrix.

	Desire to Be a Scientist		Science Possible Selves		Science Confidence		Boy-Science-Bias		Essentialist Mindsets		Fixed Mindsets		Science Grades		Minority		College Expectations		Books > 100	Grade Level
Science Possible Selves	0.37	***																		
Science Confidence	0.30	***	0.35	***																
Boy-Science-Bias	−0.01		−0.12	**	−0.03															
Essentialist Mindsets	0.06		−0.02		−0.06		0.10	*												
Fixed Mindsets	−0.05		−0.19	***	−0.15	***	0.14	**	0.26	***										
Science Grades	0.14	**	0.22	***	0.40	***	−0.18	***	−0.09	*	−0.28	**								
Minority	−0.03		−0.09	*	−0.11	*	0.06		−0.03		0.11	*	−0.12	**						
College Expectations	0.04		0.20	***	0.13	**	−0.11	*	−0.10	*	−0.13	**	0.22	***	−0.04					
Books > 100	0.05		0.11	*	0.15	***	−0.10	*	0.02		−0.05		0.13	**	−0.20	**	0.13	**		
Grade Level	0.03		0.08	+	−0.02		0.04		0.01		−0.03		−0.24	***	0.06		0.02		−0.02	
Girls	−0.08	+	−0.09	*	−0.10	**	−0.12	**	−0.04		−0.04		−0.04		0.01		0.04		−0.04	−0.04

Note:

+ p < 0.10,

* p < 0.05,

** p < 0.01,

*** p < 0.001.

**Table 3:** Logistic Regression Models Predicting Boy-Science Bias A and Science Confidence B.

	Boy-Science Bias						Science Confidence					

	Model 1			Model 2			Model 3			Model 4		

	β	SE	*p*	β	SE	*p*	β	SE	*p*	β	SE	*p*
Grade Level	0.08	0.15		−0.17	0.19		0.26	0.16	+	0.32	0.20	
Girl (Boy Reference)	−0.75	0.26	*	−0.81	0.27	*	−0.47	0.26	+	−0.48	0.26	+
GirlXGrade Level	-	-		0.72	0.33	*	-	-		−0.15	0.31	

**Focal Independent Variables**												

***Mindsets***												
Essentialist Mindsets	0.13	0.10		0.14	0.10		−0.05	0.11		−0.05	0.11	
Fixed Mindsets	0.24	0.10	*	0.24	0.11	*	−0.07	0.10		−0.07	0.10	
Boy-Science Bias							0.46	0.38		0.46	0.38	

**Controls**												

Science Grades	-	-		-	-		1.07	0.15	***	1.07	0.15	***
Racial/Ethnic Minority (White reference)	0.13	0.30		0.13	0.30		−0.18	0.26		−0.18	0.26	
***Social Capital***												
College Expectations	−0.15	0.14		−0.14	0.14		0.10	0.18		0.10	0.18	
Books in the home (0–10 reference)												
10–99 books	−0.56	0.29	*	−0.61	0.29	*	0.33	0.39		0.34	0.39	
100+ books	−1.09	0.39	**	−1.14	0.39	**	0.79	0.43	+	0.81	0.43	
intercept	−1.02	0.35	**	−1.16	0.68	**	−7.91	1.23	***	−2.24	0.46	***
Nagelkerke R squared		0.11	***		0.13	***		0.34	***		0.34	***

Notes:

A Do you think boys or girls are better at science? Predicted = “Boys are a little/lot better at science.” Reference = “Girls and boys are the same at Science,” “Girls are a little/lot better at science.”

B How good are you at Science? Predicted = “Excellent.” Reference = “Good,” “Fair,” “Poor.”

+ p < 0.10,

* p < 0.05,

** p < 0.01,

*** p < 0.001.

**Table 4:** Logistic Regression Models Predicting Possible Selves A and Desire to be a Scientist B.

	Science Possible Self						Desire to be a Scientist		

	Model 1			Model 2			Model 3		

	β	SE	*p*	β	SE	*p*	β	SE	*p*
Grade Level	0.33	0.15	*	0.33	0.15	*	0.28	0.25	
Girl (Boy Reference)	−0.40	0.24	+	−0.42	0.25	+	−0.27	0.40	

**Focal Independent Variables**									

***Mindsets***									
Essentialist Mindsets	0.12	0.09		0.11	0.09		0.25	0.15	
Fixed Mindsets	−0.28	0.11	*	−0.27	0.11	*	0.09	0.17	
Boy-Science Bias	−1.00	0.42	*	−1.04	0.47	*	0.26	0.57	
GirlXBoy-Science Bias	-	-		0.32	0.91		-	-	
Science Confidence	1.49	0.29	***	1.49	0.28	***	1.48	0.45	**
Science Possible Self	-	-		-	-		2.42	0.45	***

**Controls**									

Science Grades	0.13	0.09		0.11	0.09		0.09	0.16	
Racial/Ethnic Minority (white reference)	−0.15	0.25					−0.16	0.42	
***Social Capital***									
College Expectations	0.57	0.18	**	0.57	0.18	**	−0.21	0.25	
Books in the home (0–10 reference)									
10–99 books	−0.13	0.33		−0.13	0.33		−0.20	0.51	
100+ books	−0.04	0.38		−0.03	0.37		−0.26	0.58	
intercept	−1.27	0.38	**	−1.27	0.38	**	−4.88	1.38	***
Nagelkerke R squared		0.26	***		0.26	***		0.34	***

Notes:

A Let’s Pretend you wanted to become a scientist, could you become a scientist if you wanted to? Outcome = “I could become a scientist.” Reference = “I might be able to become scientist,” “I probably could not become a scientist,” “I could not become a scientist,” “I don’t know.”

B How much do you want to become a scientist? Outcome = “A lot.” Reference= “Some,” “A little,” “Not at all.”

+ p < 0.10,

* p < 0.05,

** p < 0.01,

*** p < 0.001.

## Footnotes

1 We chose to dichotomize our dependent variables for multiple reasons. First, the explicit gender bias scale includes a girl science bias, but we dichotomized this variable and included those with a girl-science-bias with youth who report no bias because it is likely that a girl science bias has a different meaning and interpretation for boys and girls in a society with documented cultural biases favoring boys in science. We will explore girl-science-bias more fully in future work. Second, for the desire to be a scientist, science possible selves, and science confidence, we were interested in assessing the odds that someone would fall into the highest category compared to all others. We conducted a number of sensitivity analyses to assess how our results might differ if we use OLS regression on these four categories, and ordinal variables rather than the dichotomized variables. We found that the results varied little if we used the dichotomized or ordinal analyses. Our main findings for the associations of gender with the outcomes and among the core concepts (i.e., mindsets, gender bias, science confidence, science possible selves, and the desire to be a scientist) were similar for both approaches. Associations for two of the control variables (social capital and self-reported science grades) were significant in the OLS models with the ordinal outcomes but not in the logistic regression models with the dichotomous outcome. We interpret these differences in the models as indicating that social capital and science grades matter more when distinguishing among the lower categories.
